# Advances in Molecular Genetics Enabling Studies of Highly Pathogenic RNA Viruses

**DOI:** 10.3390/v14122682

**Published:** 2022-11-30

**Authors:** Christian Mittelholzer, Thomas Klimkait

**Affiliations:** Department Biomedicine, University of Basel, Petersplatz 10, 4051 Basel, Switzerland

**Keywords:** SARS-CoV-2, HIV-1, reverse genetics, RNA viruses, cloning

## Abstract

Experimental work with viruses that are highly pathogenic for humans and animals requires specialized Biosafety Level 3 or 4 facilities. Such pathogens include some spectacular but also rather seldomly studied examples such as Ebola virus (requiring BSL-4), more wide-spread and commonly studied viruses such as HIV, and the most recent example, SARS-CoV-2, which causes COVID-19. A common characteristic of these virus examples is that their genomes consist of single-stranded RNA, which requires the conversion of their genomes into a DNA copy for easy manipulation; this can be performed to study the viral life cycle in detail, develop novel therapies and vaccines, and monitor the disease course over time for chronic virus infections. We summarize the recent advances in such new genetic applications for RNA viruses in Switzerland over the last 25 years, from the early days of the HIV/AIDS epidemic to the most recent developments in research on the SARS-CoV-2 coronavirus. We highlight game-changing collaborative efforts between clinical and molecular disciplines in HIV research on the path to optimal clinical disease management. Moreover, we summarize how the modern technical evolution enabled the molecular studies of emerging RNA viruses, confirming that Switzerland is at the forefront of SARS-CoV-2 research and potentially other newly emerging viruses.

## 1. Introduction

In the long global race for the most-suitable tools for the molecular research of viruses, the first reports appeared in the late 1970s and early 1980s, which described the successful generation of infectious cDNA clones of certain plus-strand RNA viruses ([1,2], reviewed in [3]). The fact that the in vitro-produced cDNA copies were able to initiate the production of replicating RNA viruses in bacterial or eukaryotic cell culture initially came as a surprise, especially because both insert orientations in the plasmid vector were able to support virus production. No definite explanation could be given for the underlying mechanism, but it was then speculated that a “random initiation of transcription” on the plasmidic DNA was responsible, with a subsequent end trimming of the RNA that generated the authentic full-length genomic RNA of the pathogen [2]. The in vitro insertion of bacterial promoters in the plasmid clones then enabled a targeted enzyme-driven in vitro transcription of infectious RNA from cDNA clones. However, successful expression remained complex in some cases and only indirectly worked via the generation of minus-strand RNA in a first step, followed by copying it into the plus-stranded complement, which was then infectious [4]. While still far from trivial, these successes began to make viral pathogens accessible, where the application was initially mainly for small genome sizes of about 4 kb and up to 7.5 kb.

It was exactly during this period that the truly revolutionary new molecular technique, “polymerase chain reaction (PCR)”, was invented. In the same decade, HIV as a new viral pathogen and threat to human health became known. Hence, in the 1990s, HIV turned into one of the main research targets, in which the virus developed into the cause of a new major global epidemic. Of note in this period, most sophisticated but standard methods of reverse genetics and DNA cloning were still in their infancy or simply not yet available.

Despite rapid progress in many disciplines of research, and although the first infectious HIV clones became available already in the mid-1980s [5], it was still a feat in 1996 to generate an infectious cDNA clone for classical swine fever virus with a genome length of 12.3 kb [6]. The successful technique required a PCR amplification of multiple small DNA fragments, which then had to be introduced into bacterial plasmids as intermediate vectors. Special DNA manipulation strategies were essential for avoiding the accidental misincorporation of too many nucleotides differing from the original viral sequence during the DNA cloning process.

However, since then, a true technical evolution with the discovery of highly faithful and processive thermostable enzymes made it possible to increase the length of functional PCR products from close to 2 kb originally to more than 10 kb today. Antiretroviral research, mainly on HIV-1, HIV-2, or SIV, greatly benefited from this development.

## 2. EARLY CASE: Molecular Genetics for HIV

A truly impressive wave of key inventions and new technological developments, which began during the 1980s, yielded a multitude of new tools with tremendous importance for research, modern laboratory diagnostics, and therapy. HIV was one of the initial viral targets for which the enormous technological developments advanced research and led to a rapid commercialization and certification of previously unavailable tools. The sensitive and specific detection of this pathogen became possible in various specimen types. Further on in the 1990s, quantitative PCR began to form the basis for the quantitative determination of viruses in blood and cerebrospinal fluid; Sanger-based sequencing [7] or the new high-content capillary sequencing techniques [8] up to the development of the “Next-Generation Sequencing” (NGS) protocols [9] made the discrimination and detailed characterization of circulating virus subtypes and resistant variants possible. In addition, special tools such as single-cell sequencing became available, which allowed for the analysis of infection events in individual cells in special infection situations such as early infection phases [10].

These techniques improved capabilities in the context of HIV diagnostics and greatly assisted long-term therapy concepts. In this way, HIV disease monitoring had become one of the first disciplines in virology that actively benefitted from modern molecular tools by the early 1990s, early in the globally devastating epidemic. It had only taken 11 years from the first FDA-approved serologic test in 1985 [11] to the approval of an RNA-based quantitative virus test in 1996 [12].

### 2.1. Viral Load Diagnostics for HIV

Until the early 1990s, RNA preparation and detection technologies remained very tedious. In addition, validated PCR protocols for nucleic acid amplification were still very scarce. Since then, through new agents and refinements, systems now reach the exquisite sensitivity of only a few viral genome copies per milliliter of sample volume, an impressive development since the commercialization of the first sensitive RNA-based tests [12]. Antibody- and antigen-based HIV tests were developed in the late 1980s and continue to be important standard diagnostic tools for first diagnosis. However, the high sensitivity of viral RNA tests proved to be more versatile, addressing a critical need in clinical situations where systems for earliest detection are essential; the tests covered situations such as after accidental exposure (US CDC [13]), during pregnancy (US guidelines [14]), or post-exposure situations (In Switzerland: *Schweiz Med Forum* 2014; 14(8): 151–153). Single-copy detection of viral RNA in a given sample often provide critical information before specific antibodies are produced in the affected individual [15].

In the very early years of the HIV/AIDS epidemic, in 1988, Swiss clinical experts had founded the “Swiss HIV Cohort Study” (SHCS). This study, mainly based on clinical and epidemiological aspects of a newly emerged disease, was led by clinicians of the university hospitals of Basel, Bern, Geneva, Lausanne, and Zurich, and of the cantonal hospital St. Gallen. The systematic collection and archiving of clinical specimens and the associated information about how to treat the “new disease” of AIDS made numerous projects on fundamental HIV research possible. Countless studies resulted, involving HIV resistance, virus evolution during and aside from therapy, causes and bystanders of therapy failure, and a multitude of highly relevant disease and treatment aspects. From 1988 until today, the SHCS is making continuous developments and has become one of the top research consortia worldwide in the field of HIV/AIDS research and therapy [16,17]. A number of milestones with global importance and impact mark its path of success. Here, we highlight only a few aspects from among its publications, which number greater than 1000.

### 2.2. The Swiss Statement on Transmission

It should be noted that it is only through the availability and use of genetic tools in clinical diagnostics that many of the impressive developments in clinical practice have become possible. In 2008, the “Swiss Statement”, key for an important stress relief of many infected persons, was coined and published by Vernazza, Hirschel, Bernasconi, and Flepp [18], today translated into “U equals U” (undetectable equals untransmissible). The study demonstrated that in a stable sexual relationship and regular intake of the antiretroviral medication, the absence of a detectable virus in the blood serves as a reliable marker for no risk of virus transmission. Without the ability to quantify low amounts of circulating viruses to below 50–100 copies/mL, the prediction would not have been possible.

Internationally, this study had quite a bumpy start in the field, as it was initially fiercely disputed as an unethical simplification of HIV management. However, in 2022, it has become the generally accepted standard for successful long-term HIV therapy.

### 2.3. Testing Therapy-Resistance of HIV

Other key milestones in the field emerged from top molecular research work, e. g., in the area of viral resistance, where Geneva researchers established viral resistance testing early on, already before 1997 [19,20]. The group of Luc Perrin and others used virus-derived genomic RNA, which was converted to cDNA and amplified the drug target regions of the viral genome, i.e., reverse transcriptase and protease. This information could then be used to determine new mutations in the virus genome, which differ from the known consensus sequence. Research groups from Geneva, Basel, and Zurich along with many others established the fact that incomplete virus suppression often correlates with detectable mutations in the target genes for the drugs taken by the affected individual ([21]; review in [22]). This knowledge allowed for the development of prediction tools, which associated specific mutations with the failure risk of certain drugs and classes as well as predictive genotype-based databases and guidelines [23,24,25]. Today, gene sequences and viral isolates are regularly collected and updated in publications such as the comprehensive compendium of the Los Alamos laboratory [26].

### 2.4. Structured Treatment Interruption

Increasing the survival time of persons living with HIV and improving drugs and regimens in the context of a life-long therapy created the growing need and demand for therapeutic simplification schemes in clinical practice. Additionally in this context, the availability of highly sensitive virus monitoring became a critical element. An example of a successful reduction of the pill and drug burden for treated individuals required regular blood sampling for the molecular characterization of any emerging virus population. One key clinical question of the interruption study was: would a continuous suppression of HIV during treatment to below 20 virus copies per milliliter of blood safely allow (short) for episodes of completely stopping therapy? Viral loads and emerging resistance mutations were monitored in free virus or proviral DNA in infected circulating cells. Despite some initially exciting hints of success [27], most important studies towards such a strategy of simplifying HIV combination therapy led to the sobering conclusion that a therapy interruption was not advisable, as reviewed by Oxenius and Hirschel [28] and analyzed by Yerly et al. [29].

### 2.5. Therapy Simplification

Furthermore, other approaches have been based on the long-term stable situation with a continuous and complete virus suppression in blood as the starting point for the simplification of the standard triple therapy for HIV. In this context, molecular genetics and the ability to detect single infected cells and minorities of drug-resistant variants in clinical specimens turned out to be as instrumental so as to be crucial. Various clinical simplification studies were conducted, which reduced the regimen to only one drug class. Meanwhile, some studies, especially for the integrase inhibitor class, suggested that indeed such a simplification can be safe and successful for certain individuals; this did not lead to a general treatment recommendation, as most studies resulted in an inferiority of the simplification arm. Other studies, mainly on the class of protease inhibitors, demonstrated the important caveat that besides blood, the brain/cerebrospinal fluid can also serve as the site of active HIV replication, as in the ATARITMO study by Vernazza et al. [30]. Consequently, full virus suppression may not be reliably reached when simplifying to certain antiretroviral drugs.

### 2.6. Approaches to Eliminate/Cure HIV

Today, we review a long era of successful HIV treatment. This success is largely based on numerous important studies which now define the optimal therapy start (from “CD4 below 200” to “test-and-treat”) or identify any best regimens for a successful long-term treatment. In recent years, an entirely new chapter was opened, with recently developed molecular tools allowing for the single-cell analysis of viral gene replication, RNA-sequencing techniques for detecting viral activity in individual cells in clinical specimens, and refined in vitro methods for revealing viral intactness and virus outgrowth.

On the clinical side of an “optimal disease management”, HIV disease principally became a “stable chronic disease”, leading to a normal quality of life. The chronicity of the retroviral infection leads to a very high stability of the mostly latent HIV genomes. Clinically, this means that a favorable clinical course is only maintained so long as antiretroviral therapy suppresses the virus. As soon as the therapy situation becomes suboptimal, the virus begins to replicate again. The recognition of this special property opened completely new research directions towards novel solutions.

One of these trigger points was the clinical description of the “Berlin-patient” case, in whom HIV could be successfully eliminated after stem cell transplantation [31]. Key findings on the clinical properties of this case triggered research approaches aiming to find ways for curing HIV through an active and complete elimination of HIV from the body of an infected person.

At the same time, new molecular tools became available that now allow for the assessment of single-cell infection events. The unique genome accession and gene targeting that became possible by utilizing the Crispr/Cas9 system also created new ideas for approaching HIV elimination, e.g., Martins et al.’s review attempts to modify the CCR5 chemokine receptor by using Crispr/Cas [32] and the Swiss Zurich group’s suggestion to target, e.g., the HIV promoter region within the viral long terminal repeat [33]. Nevertheless, these new techniques have not yet been able to influence clinical disease management.

Highly sensitive cell diagnostic tools were developed, where Swiss research again provided crucial contributions. One of the big developments came from the discovery and molecular characterization of broadly neutralizing antibodies that may be present in infected individuals. The unique property of these specific antibodies, which mostly target the viral envelope, is that they can elicit an immune response that allows for the neutralization of a broad range of HIV isolates and subtypes [34,35]. For possible clinical applications in the future, the hope is that these antibodies can either be used for new antiviral approaches or to provoke a similar kind of immune response to de novo in an infected individual.

Swiss research contributions to other specific cure approaches include the targeted interference with the ongoing immune response of the infected human host. Specifically, the combination of strategies that stimulate the immune defense on the one hand and are aimed at reducing or eliminating replication-competent HIV on the other show promise (reviewed by Perreau et al. 2017 [36]).

### 2.7. International Development Work for HIV Therapy and Diagnostics

Beyond the ground-breaking basic and clinical scientific research work, clinics in several African countries, including Tanzania, Uganda, Lesotho, and others, have critically benefitted from international collaborations with Swiss experts. Among many truly game-changing clinical advancements in the daily clinical work of patient management, the “import” and establishment of state-of-the-art “reverse genetics” tools was critical. The provision of sequence analyzer instruments or new laboratory diagnostic techniques and a strong influx of knowledge helped to completely change the hopeless therapy perspective of the 1990s into a new century of hope for HIV/AIDS-affected persons.

Initially, the grim truth in this context had been phrased by a plenary speaker at the International AIDS Conference in the early 1990s as “Africa is gone”. The desperate therapy situation (UNAIDS report of 1998 [37]) was transformed by the rapid response of philanthropes, industry, and research, a blessing and true game-changer for an entire continent and beyond. The financially potent contributors include(d) public and private funders through amfAR, the Global Fund, UNAIDS, the US PEPFAR, the Gates Foundation, CHAI, and numerous others.

On the basis of the technical level alone, therapy-guiding resistance testing for HIV has also now become part of the clinical routine in Africa and other resource-limited regions, which has helped to transform standard therapy in collaborating centers, where experts from Switzerland and other countries train national physicians and laboratory personnel [38,39,40,41]. This important work became recognized up to the level of the WHO for international guidelines [42]. Our active contributions, creative molecular work, modern genetic studies, and capacity building continue to evolve in clinical and research centers abroad, which importantly contributes to providing direct benefits for human health.

## 3. LATEST CASE: Reverse Genetics for Coronaviruses

The more recent members of pathogenic RNA viruses, the human coronaviruses such as SARS-CoV-2, possess the largest genomes of all known RNA viruses, with about a 30 kb length for a single ribonucleic acid element. This causes any purely PCR- or plasmid-based approach for genome manipulation to be very technically challenging. As a consequence, the inherent need to clone very large DNA fragments along with the presence of sequence stretches that seem to be toxic for cloning or expression in bacteria prevented the establishment of functional cDNA clones of coronaviruses for quite some time. In addition, the pandemic potential that is associated with the more recently emerged new viruses with a high pathogenic potential for humans, such as SARS-1, MERS, or SARS-CoV-2, necessitated the use of a BSL-3 environment for their manipulation in vitro. This fact massively limited and continues to limit molecular and viral research, making work with these viruses very laborious.

On the other hand, the technical advancements of recent years, which include the provision of numerous commercially available research tools such as high-fidelity enzymes and reagents or complete cloning kits has enabled the present generation of rather error-free, infectious cDNA clones in a single piece and has yielded the production of RNA viruses with a genome size of up to 15 kb in vitro. As a result, in recent years, even larger virus genomes have become and will continue to become readily accessible for research and development.

### 3.1. BAC-Cloning

The first complete infectious cDNA clone for a coronavirus was only reported in 2000 by Almazan et al. [43], who employed a bacterial artificial chromosome (BAC). While BACs allow for the cloning of the full-length cDNA copy in a single piece without the instabilities of inserted sequences, which is frequently observed after cloning into (high copy number) plasmids, the low copy number of one or two copies per cell poses other significant challenges. BACs routinely need to be purified by CsCl centrifugation to obtain highly pure DNA in sufficient amounts for further applications, e.g., for in vitro transcription. In addition, BACs must have the insertion of an independent eukaryotic promoter element such as a CMV promoter in front of the viral sequence to allow for the direct transfection of DNA into cells without the need for first transcribing it in vitro into full-length viral RNA before the nuclease-sensitive RNA can be transfected into cells.

### 3.2. In Vitro Assembly and Vaccinia-Vectored Approaches

As an alternative approach, the cell-free assembly of full-length cDNA copies in vitro was established at about the same time [44]. Furthermore, new vaccinia-vectored approaches were described for the generation of intact, functional coronavirus genomes [45]. Details of these techniques and their uses have been reviewed in detail by Almazan et al. in 2014 [46]; the details were of great utility for work with the newly emerging human-pathogenic coronaviruses SARS-CoV-1 and MERS and gave molecular coronavirus research an important boost. Common to all above-mentioned techniques and similar to the example of the classical swine fever virus, the availability or introduction of suitable restriction sites was key, which permitted specifically cutting PCR-generated fragments and ligating them in a sequence-precise fashion. Such carefully designed strategies often depend on unique sequences only present in a certain coronavirus species or in a specific strain of interest and hence lack the versatility needed when studying many different strains, variants, or even newly emerging viruses. In addition, the generation of sufficient quantities of faithfully copied, error-free full-length RNA transcripts to allow for the efficient rescue of infectious virus after cell introduction poses another major challenge. For this, it is critical that the DNA template is of high quality, and the reaction conditions need to be optimal to achieve sufficient yields of the full-length transcripts. Moreover, productively transfecting long RNA molecules into cells is more often a case of trial and error than being easy and predictable.

### 3.3. TAR Cloning and CPER

More recently, transformation-associated recombination (TAR) cloning in yeast has been developed and used for the generation of the first cDNA clone of SARS-CoV-2 [47]. This method relies on the inherent ability of the yeast cell to recombine several overlapping DNA fragments into one long product, performing this with high accuracy and efficiency. The advantage of this method is that suitable restriction sites for end trimming are no longer needed. However, the method still involves the standard in vitro transcription and RNA transfection steps for rescuing the virus.

Very recently, the circular polymerase extension reaction (CPER) was established and described for the generation of full-length cDNA clones of coronaviruses [48]. This method is based on the use of overlapping fragments, which can assemble without the need for unique restriction sites. The CPER technique is based on an in vitro assembly in the test tube, where long cDNA fragments self-assemble into a transfectable DNA product. The product is designed in a way such that it is driven by a heterologous (CMV) promoter at the 5’ end to transcribe infectious RNA after DNA transfection into the target cell. While the circular nature of the product might confer a higher stability inside the transfected cell, the principal step of transfecting such large DNA molecules remains far from trivial. This may explain why Torii et al. [48], despite successfully using a variety of CPER conditions for viral rescue, could only generate the virus exclusively in HEK293-3P6C33 cells, which are known for their superb transfectability.

### 3.4. ISA

**Figure 1:**
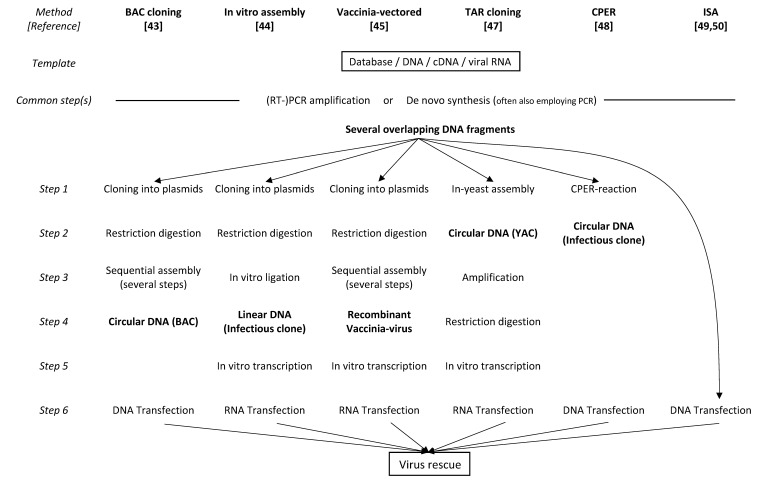
Comparison of major technologies for rescuing coronaviruses.

Another recently described DNA-based method, the ISA (Infectious Subgenomic Amplicons) technique, was originally developed using small RNA viruses, such as dengue virus and chikungunya virus [49]. This method has now been adapted to also address SARS-CoV-2 [50]. After the simultaneous introduction of eight overlapping DNA fragments constituting the entire viral genome, the authors were able to directly rescue the infectious virus in susceptible cells. As a discriminating feature, the ISA technique is based on the cellular introduction of subgenomic DNA segments rather than RNA. The intentional use of subgenomic DNA facilitates a more efficient transfection due to the smaller size of individual DNA. The intracellular reassembly of the viral DNA segments reconstituting a complete viral genome is guided by overlaps of the DNA segments and spontaneously occurs inside the target cell. Therefore, this method completely circumvents any need for restriction sites in the in vitro transcription of long RNA molecules and in the transfection of long RNAs into cells. A comparison of the above-mentioned coronavirus reverse genetics methods is shown in Figure 1.

### 3.5. Role of N Protein in Virus Recovery

In principle, genomic RNA of a plus-strand RNA virus is all that is needed to start the generation of virus progeny inside a host cell. Interestingly, most if not all recovery approaches for functional SARS-CoV-2 [47,48,51] and other coronaviruses [44,52,53,54] involve a co-transfection of N (nucleocapsid) mRNA or an expression plasmid for N to enhance virus recovery. While the viral genome is covered with N in a natural infection, the RNA transcribed from the introduced DNA by the cellular RNA polymerase [43,48,50] or transfected as naked, in vitro transcribed RNA [44,45,47] lacks this protection and might be more vulnerable to degradation before the replication process is initiated. It is interesting to see whether the additional N is an absolute technical requirement for SARS-CoV-2 rescue in vitro or if an efficient system can be established that circumvents the need for this additional component, which would confirm that genomic RNA is all it takes to start virus replication.

### 3.6. Role of the Polybasic S1/S2 Cleavage Site in the S Protein

While control of virus variants is easily possible down to single nucleotides on the DNA level by establishing clonal copies of the respective gene or region, matters tend to get more complicated when RNA needs to be assessed. This is due to the rather high inherent error rates of RNA polymerases and because direct RNA cloning is not available; any manipulation prior to analysis involves another one of these error-prone steps.

The evolutionary acquisition of a polybasic S1/S2 cleavage site in the spike protein of SARS-CoV-2 most likely represents an essential activation step and is possibly necessary for allowing the virus to establish itself successfully in the (new) host. The fact that virtually all human isolates of SARS-CoV-2 possess this cleavage site indicates that this function is indispensable for proper replication in the human host. However, when the standard cell model of VeroE6 cells is used for virus propagation, this cleavage site tends to get very rapidly lost and is even selected against within only a handful of cell culture passages in VeroE6 cells. The observation that even an obviously critical protein domain in a viral protein that is essential for the virus in vivo can change or get lost in a cell model is a big alert to the non-critical use of cell lines in vitro. The loss of the S1/S2 cleavage site was also shown to have significant functional consequences, such as altering the transmissibility in ferrets, changing the pathogenicity in animal models, and the ability of the virus to use endosome-independent virus entry by a route that avoids antiviral IFITM proteins [55,56,57].

### 3.7. Cell and Animal Models for SARS-CoV-2 Infection

Cleavage at the polybasic S1/S2 site upon binding of the SARS-CoV-2 protein site to its receptor is an important first step in the infection process. The presence of the human TMPRSS2 protease has been shown to efficiently activate the spike protein of SARS-CoV-1 [58] and enhance the in vitro production of SARS-CoV-1, MERS-CoV, and SARS-CoV-2 in VeroE6 cells [59]. In addition, the expression of TMPRSS2 in VeroE6 cells leads to the stable retention of the sequence for the polybasic S1/S2 cleavage site in the SARS-CoV-2 spike protein, even after more than 10 in vitro passages ([60,61]). Using TMPRSS2-expressing cells seems to be imperative for keeping these human betacoronaviruses in a state that is close to the natural situation.

VeroE6 cells are derived from the kidney of the African green monkey (Chlorocebus sp.) and therefore also display an angiotensin that converts the enzyme 2 (ACE2) molecule, the receptor for SARS-CoV-2 and SARS-CoV-1, in the version of a non-human primate. Even though the very same RBD-binding amino acids are conserved between humans and monkeys, the monkey ACE2 slightly differs from the human ACE2 [62,63]. This emphasizes that these cells, which are commonly used due to their property of showing a clearly visible cytopathic effect upon viral infection, should be used with some caution [64].

While animal models are key for research and vaccine development, the example of SARS-CoV-2 may be quite instructive again. On the one hand, newer models such as those using the Syrian golden hamster or ferrets may not perfectly mirror the situation in humans among others due to slight differences in the ACE2 receptor molecule or in the proteases involved in the cleavage of the viral spike protein. However, they are widely used, as the infection and affected organs correlate rather well with the pathology seen in humans. On the other hand, experimental mouse models, which express a human ACE2 molecule and can be infected with SARS-CoV-2 seem to be a good system for assessing the immunological response. Nevertheless, it should be noted that the K18-ACE2 transgenic mouse model represents one of the few in vivo models where an acute SARS-CoV-2 infection leads to severe brain pathology and death [65]. This phenotype is very uncommon in humans and requires further detailed studies of the ill-understood link between SARS-CoV-2 and brain infection in mice. This feature could reflect a serious limitation, potentially even questioning the K18-ACE2 model as a predictive disease and vaccination model.

### 3.8. Perspectives for Next-Generation Vaccines

Fundamental studies on coronavirus replication and individual protein functions using mutants with gene deletions have revealed that the deletion of individual structural genes may generate both attenuated and propagation-defective viruses, which have the potential to be developed into vaccine candidates. This has been shown for SARS-CoV-1 [66,67,68,69,70,71,72] and MERS-CoV [73,74,75]. Recently, deletion mutants and trans-complementation systems have also been established for SARS-CoV-2 [76,77,78] and have opened the perspective of developing safe and effective next-generation SARS-CoV-2 vaccines. The greatest advantage of such infectious, single-cycle SARS-CoV-2 viruses is that they are able to present all structural viral proteins to the immune system to induce humoral responses, and upon infection, the host cells can produce all the nonstructural viral proteins to induce cell-mediated immune responses.

## 4. Conclusions

The molecular aspects of HIV research nicely summarize how a rapid incorporation of new developments and advances in molecular genetics into diagnostics and medical disease management is possible; today, this can occur with an amazing translational speed. Superb collaborative efforts between clinical and molecular disciplines were and still are instrumental for ensuring rapid and solid progress, not only in the fight against HIV, but also in response to the newest advances against the very recent COVID-19 pandemic.

With this line of successful developments, the scientific landscape in Switzerland shows a robust potential for the very rapid development and implementation of epidemiologic and diagnostic tools and research towards novel therapies and vaccines, both against well-known and emerging viruses. Moreover, these developments, driven by modern molecular genetics, may at the same time serve as strong indicators for our preparedness for new pathogenic epidemics or pandemics to come.

## References

[1] Taniguchi T., Palmieri M., Weissmann C. (1978). QB DNA-containing hybrid plasmids giving rise to QB phage formation in the bacterial host. Nature.

[2] Racaniello V.R., Baltimore D. (1981). Cloned poliovirus complementary DNA is infectious in mammalian cells. Science.

[3] Boyer J.C., Haenni A.L. (1994). Infectious transcripts and cDNA clones of RNA viruses. Virology.

[4] Kaplan G., Lubinski J., Dasgupta A., Racaniello V.R. (1985). In vitro synthesis of infectious poliovirus RNA. Proc. Natl. Acad. Sci. USA.

[5] Adachi A., Gendelman H.E., Koenig S., Folks T., Willey R., Rabson A., Martin M.A. (1986). Production of acquired immunodeficiency syndrome-associated retrovirus in human and nonhuman cells transfected with an infectious molecular clone. J. Virol..

[6] Ruggli N., Tratschin J.D., Mittelholzer C., Hofmann M.A. (1996). Nucleotide sequence of classical swine fever virus strain Alfort/187 and transcription of infectious RNA from stably cloned full-length cDNA. J. Virol..

[7] Sturmer M., Reinheimer C. (2012). Description of two commercially available assays for genotyping of HIV-1. Intervirology.

[8] Merel P., Pellegrin I., Garrigue I., Caumont A., Schrive M.H., Birac V., Bonot P., Fleury H. (2001). Comparison of capillary electrophoresis sequencing with the new CEQ 2000 DNA Analysis System to conventional gel based systems for HIV drug resistance analysis. J. Virol. Methods.

[9] Gall A., Ferns B., Morris C., Watson S., Cotten M., Robinson M., Berry N., Pillay D., Kellam P. (2012). Universal amplification, next-generation sequencing, and assembly of HIV-1 genomes. J. Clin. Microbiol..

[10] Henn M.R., Boutwell C.L., Charlebois P., Lennon N.J., Power K.A., Macalalad A.R., Berlin A.M., Malboeuf C.M., Ryan E.M., Gnerre S. (2012). Whole genome deep sequencing of HIV-1 reveals the impact of early minor variants upon immune recognition during acute infection. PLoS Pathog..

[11] (1985). Provisional Public Health Service inter-agency recommendations for screening donated blood and plasma for antibody to the virus causing acquired immunodeficiency syndrome. MMWR Morb. Mortal. Wkly Rep..

[12] Saag M.S., Holodniy M., Kuritzkes D.R., O’Brien W.A., Coombs R., Poscher M.E., Jacobsen D.M., Shaw G.M., Richman D.D., Volberding P.A. (1996). HIV viral load markers in clinical practice. Nat. Med..

[13] CDC information Post-Exposure Prophylaxis (PEP) | HIV Risk and Prevention. https://www.cdc.gov/hiv/risk/pep/index.html.

[14] Recommendations for the Use of Antiretroviral Drugs During Pregnancy and Interventions to Reduce Perinatal HIV Transmission in the United States. https://clinicalinfo.hiv.gov/en/guidelines/perinatal/intrapartum-care-for-people-with-hiv?view=full.

[15] Lee B.E., Plitt S.S., Jayaraman G.C., Chui L., Singh A.E., Preiksaitis J.K. (2012). Use of quantitative HIV RNA detection for early diagnosis of HIV infection in infants and acute HIV infections in Alberta, Canada. J. Clin. Microbiol..

[16] Ledergerber B., von Overbeck J., Egger M., Luthy R. (1994). The Swiss HIV Cohort Study: Rationale, organization and selected baseline characteristics. Soz. Praventivmed..

[17] Swiss HIV Cohort Study Information. https://www.shcs.ch/157-about-shcs.

[18] Swiss Statement on Transmission. https://saez.ch/journalfile/view/article/ezm_saez/fr/bms.2008.13252/ceec6bc5af6bc91092a76b506e46e296f6dfc5f7/bms_2008_13252.pdf/rsrc/jf.

[19] Lorenzi P., Yerly S., Abderrakim K., Fathi M., Rutschmann O.T., von Overbeck J., Leduc D., Perrin L., Hirschel B. (1997). Toxicity, efficacy, plasma drug concentrations and protease mutations in patients with advanced HIV infection treated with ritonavir plus saquinavir. Swiss HIV Cohort Study. AIDS.

[20] Lorenzi P., Opravil M., Hirschel B., Chave J.P., Furrer H.J., Sax H., Perneger T.V., Perrin L., Kaiser L., Yerly S. (1999). Impact of drug resistance mutations on virologic response to salvage therapy. Swiss HIV Cohort Study. AIDS.

[21] von Wyl V., Klimkait T., Yerly S., Nicca D., Furrer H., Cavassini M., Calmy A., Bernasconi E., Boni J., Aubert V. (2013). Adherence as a predictor of the development of class-specific resistance mutations: The Swiss HIV Cohort Study. PLoS ONE.

[22] Hauser A., Goldstein F., Reichmuth M.L., Kouyos R.D., Wandeler G., Egger M., Riou J. (2022). Acquired HIV drug resistance mutations on first-line antiretroviral therapy in Southern Africa: Systematic review and Bayesian evidence synthesis. J. Clin. Epidemiol..

[23] Tang M.W., Liu T.F., Shafer R.W. (2012). The HIVdb system for HIV-1 genotypic resistance interpretation. Intervirology.

[24] Euresist Network A European Multidisciplinary Network for the Fight against HIV Drug Resistance. https://www.euresist.org.

[25] EACS (European AIDS Clinical Society) Guidelines. https://www.eacsociety.org/guidelines/eacs-guidelines/.

[26] HIV Databases at Los Alamos National Laboratory. https://www.hiv.lanl.gov/content/index.

[27] Papasavvas E., Kostman J.R., Mounzer K., Grant R.M., Gross R., Gallo C., Azzoni L., Foulkes A., Thiel B., Pistilli M. (2004). Randomized, controlled trial of therapy interruption in chronic HIV-1 infection. PLoS Med..

[28] Oxenius A., Hirschel B. (2003). Structured treatment interruptions in HIV infection: Benefit or disappointment?. Expert Rev. Anti. Infect Ther..

[29] Yerly S., Fagard C., Günthard H.F., Hirschel B., Perrin L. (2003). Drug resistance mutations during structured treatment interruptions. Antivir. Ther..

[30] Vernazza P., Daneel S., Schiffer V., Decosterd L., Fierz W., Klimkait T., Hoffmann M., Hirschel B. (2007). The role of compartment penetration in PI-monotherapy: The Atazanavir-Ritonavir Monomaintenance (ATARITMO) Trial. AIDS.

[31] Hutter G., Nowak D., Mossner M., Ganepola S., Mussig A., Allers K., Schneider T., Hofmann J., Kucherer C., Blau O. (2009). Long-term control of HIV by CCR5 Delta32/Delta32 stem-cell transplantation. N. Engl. J. Med..

[32] Hartley O., Martins E., Scurci I. (2018). Preventing HIV transmission through blockade of CCR5: Rationale, progress and perspectives. Swiss. Med. Wkly.

[33] Klinnert S., Chemnitzer A., Rusert P., Metzner K.J. (2022). Systematic HIV-1 promoter targeting with CRISPR/dCas9-VPR reveals optimal region for activation of the latent provirus. J. Gen. Virol..

[34] Rusert P., Kouyos R.D., Kadelka C., Ebner H., Schanz M., Huber M., Braun D.L., Hozé N., Scherrer A., Magnus C. (2016). Determinants of HIV-1 broadly neutralizing antibody induction. Nat. Med..

[35] Kouyos R.D., Rusert P., Kadelka C., Huber M., Marzel A., Ebner H., Schanz M., Liechti T., Friedrich N., Braun D.L. (2018). Tracing HIV-1 strains that imprint broadly neutralizing antibody responses. Nature.

[36] Perreau M., Banga R., Pantaleo G. (2017). Targeted Immune Interventions for an HIV-1 Cure. Trends Mol. Med..

[37] Report on the Global HIV/AIDS Epidemic. https://data.unaids.org/pub/report/1998/19981125_global_epidemic_report_en.pdf.

[38] Masimba P., Kituma E., Klimkait T., Horvath E., Stoeckle M., Hatz C., Mossdorf E., Mwaigomole E., Khamis S., Jullu B. (2013). Prevalence of drug resistance mutations and HIV type 1 subtypes in an HIV type 1-infected cohort in rural Tanzania. AIDS Res. Hum. Retrovir..

[39] Muri L., Gamell A., Ntamatungiro A.J., Glass T.R., Luwanda L.B., Battegay M., Furrer H., Hatz C., Tanner M., Felger I. (2017). Development of HIV drug resistance and therapeutic failure in children and adolescents in rural Tanzania: An emerging public health concern. AIDS.

[40] von Braun A., Sekaggya-Wiltshire C., Bachmann N., Ssemwanga D., Scherrer A.U., Nanyonjo M., Kapaata A., Kaleebu P., Gunthard H.F., Castelnuovo B. (2018). HIV-1 Drug Resistance Among Ugandan Adults Attending an Urban Out-Patient Clinic. J. Acquir. Immune Defic. Syndr..

[41] Amstutz A., Brown J.A., Ringera I., Muhairwe J., Lejone T.I., Klimkait T., Glass T.R., Labhardt N.D. (2020). Engagement in Care, Viral Suppression, Drug Resistance, and Reasons for Nonengagement After Home-Based Same-Day Antiretroviral Therapy Initiation in Lesotho: A Two-Year Follow-up of the CASCADE Trial. Clin. Infect. Dis..

[42] Labhardt N.D., Ringera I., Lejone T.I., Klimkait T., Muhairwe J., Amstutz A., Glass T.R. (2018). Effect of Offering Same-Day ART vs Usual Health Facility Referral During Home-Based HIV Testing on Linkage to Care and Viral Suppression Among Adults With HIV in Lesotho: The CASCADE Randomized Clinical Trial. JAMA J. Am. Med. Assoc..

[43] Almazan F., Gonzalez J.M., Penzes Z., Izeta A., Calvo E., Plana-Duran J., Enjuanes L. (2000). Engineering the largest RNA virus genome as an infectious bacterial artificial chromosome. Proc. Natl. Acad. Sci. USA.

[44] Yount B., Curtis K.M., Baric R.S. (2000). Strategy for systematic assembly of large RNA and DNA genomes: Transmissible gastroenteritis virus model. J. Virol..

[45] Thiel V., Herold J., Schelle B., Siddell S.G. (2001). Infectious RNA transcribed in vitro from a cDNA copy of the human coronavirus genome cloned in vaccinia virus. J. Gen. Virol..

[46] Almazan F., Sola I., Zuniga S., Marquez-Jurado S., Morales L., Becares M., Enjuanes L. (2014). Coronavirus reverse genetic systems: Infectious clones and replicons. Virus Res..

[47] Thi Nhu Thao T., Labroussaa F., Ebert N., V’Kovski P., Stalder H., Portmann J., Kelly J., Steiner S., Holwerda M., Kratzel A. (2020). Rapid reconstruction of SARS-CoV-2 using a synthetic genomics platform. Nature.

[48] Torii S., Ono C., Suzuki R., Morioka Y., Anzai I., Fauzyah Y., Maeda Y., Kamitani W., Fukuhara T., Matsuura Y. (2021). Establishment of a reverse genetics system for SARS-CoV-2 using circular polymerase extension reaction. Cell Rep..

[49] Aubry F., Nougairède A., de Fabritus L., Querat G., Gould E.A., de Lamballerie X. (2014). Single-stranded positive-sense RNA viruses generated in days using infectious subgenomic amplicons. J. Gen. Virol..

[50] Melade J., Piorkowski G., Touret F., Fourie T., Driouich J.S., Cochin M., Bouzidi H.S., Coutard B., Nougairede A., de Lamballerie X. (2022). A simple reverse genetics method to generate recombinant coronaviruses. EMBO Rep..

[51] Xie X., Muruato A., Lokugamage K.G., Narayanan K., Zhang X., Zou J., Liu J., Schindewolf C., Bopp N.E., Aguilar P.V. (2020). An Infectious cDNA Clone of SARS-CoV-2. Cell Host Microbe.

[52] Casais R., Thiel V., Siddell S.G., Cavanagh D., Britton P. (2001). Reverse genetics system for the avian coronavirus infectious bronchitis virus. J. Virol..

[53] Yount B., Denison M.R., Weiss S.R., Baric R.S. (2002). Systematic assembly of a full-length infectious cDNA of mouse hepatitis virus strain A59. J. Virol..

[54] Yount B., Curtis K.M., Fritz E.A., Hensley L.E., Jahrling P.B., Prentice E., Denison M.R., Geisbert T.W., Baric R.S. (2003). Reverse genetics with a full-length infectious cDNA of severe acute respiratory syndrome coronavirus. Proc. Natl. Acad. Sci. USA.

[55] Peacock T.P., Goldhill D.H., Zhou J., Baillon L., Frise R., Swann O.C., Kugathasan R., Penn R., Brown J.C., Sanchez-David R.Y. (2021). The furin cleavage site in the SARS-CoV-2 spike protein is required for transmission in ferrets. Nat. Microbiol..

[56] Johnson B.A., Xie X., Bailey A.L., Kalveram B., Lokugamage K.G., Muruato A., Zou J., Zhang X., Juelich T., Smith J.K. (2021). Loss of furin cleavage site attenuates SARS-CoV-2 pathogenesis. Nature.

[57] Hossain M.G., Tang Y.D., Akter S., Zheng C. (2022). Roles of the polybasic furin cleavage site of spike protein in SARS-CoV-2 replication, pathogenesis, and host immune responses and vaccination. J. Med. Virol..

[58] Matsuyama S., Nagata N., Shirato K., Kawase M., Takeda M., Taguchi F. (2010). Efficient activation of the severe acute respiratory syndrome coronavirus spike protein by the transmembrane protease TMPRSS2. J. Virol..

[59] Matsuyama S., Nao N., Shirato K., Kawase M., Saito S., Takayama I., Nagata N., Sekizuka T., Katoh H., Kato F. (2020). Enhanced isolation of SARS-CoV-2 by TMPRSS2-expressing cells. Proc. Natl. Acad Sci. USA.

[60] Liu Y., Zhang X., Liu J., Xia H., Zou J., Muruato A.E., Periasamy S., Kurhade C., Plante J.A., Bopp N.E. (2022). A live-attenuated SARS-CoV-2 vaccine candidate with accessory protein deletions. Nat. Commun..

[61] Amarilla A.A., Sng J.D.J., Parry R., Deerain J.M., Potter J.R., Setoh Y.X., Rawle D.J., Le T.T., Modhiran N., Wang X. (2021). A versatile reverse genetics platform for SARS-CoV-2 and other positive-strand RNA viruses. Nat. Commun..

[62] Piplani S., Singh P.K., Winkler D.A., Petrovsky N. (2021). In silico comparison of SARS-CoV-2 spike protein-ACE2 binding affinities across species and implications for virus origin. Sci. Rep..

[63] Ma C., Gong C. (2021). ACE2 models of frequently contacted animals provide clues of their SARS-CoV-2 S protein affinity and viral susceptibility. J. Med. Virol..

[64] Funnell S.G.P., Afrough B., Baczenas J.J., Berry N., Bewley K.R., Bradford R., Florence C., Duff Y.L., Lewis M., Moriarty R.V. (2021). A cautionary perspective regarding the isolation and serial propagation of SARS-CoV-2 in Vero cells. NPJ Vaccines.

[65] Dedoni S., Avdoshina V., Camoglio C., Siddi C., Fratta W., Scherma M., Fadda P. (2022). K18- and CAG-hACE2 Transgenic Mouse Models and SARS-CoV-2: Implications for Neurodegeneration Research. Molecules.

[66] DeDiego M.L., Alvarez E., Almazan F., Rejas M.T., Lamirande E., Roberts A., Shieh W.J., Zaki S.R., Subbarao K., Enjuanes L. (2007). A severe acute respiratory syndrome coronavirus that lacks the E gene is attenuated in vitro and in vivo. J. Virol..

[67] Dediego M.L., Pewe L., Alvarez E., Rejas M.T., Perlman S., Enjuanes L. (2008). Pathogenicity of severe acute respiratory coronavirus deletion mutants in hACE-2 transgenic mice. Virology.

[68] Lamirande E.W., DeDiego M.L., Roberts A., Jackson J.P., Alvarez E., Sheahan T., Shieh W.J., Zaki S.R., Baric R., Enjuanes L. (2008). A live attenuated severe acute respiratory syndrome coronavirus is immunogenic and efficacious in golden Syrian hamsters. J. Virol..

[69] Netland J., DeDiego M.L., Zhao J., Fett C., Alvarez E., Nieto-Torres J.L., Enjuanes L., Perlman S. (2010). Immunization with an attenuated severe acute respiratory syndrome coronavirus deleted in E protein protects against lethal respiratory disease. Virology.

[70] Fett C., DeDiego M.L., Regla-Nava J.A., Enjuanes L., Perlman S. (2013). Complete protection against severe acute respiratory syndrome coronavirus-mediated lethal respiratory disease in aged mice by immunization with a mouse-adapted virus lacking E protein. J. Virol..

[71] Jimenez-Guardeno J.M., Regla-Nava J.A., Nieto-Torres J.L., DeDiego M.L., Castano-Rodriguez C., Fernandez-Delgado R., Perlman S., Enjuanes L. (2015). Identification of the Mechanisms Causing Reversion to Virulence in an Attenuated SARS-CoV for the Design of a Genetically Stable Vaccine. PLoS Pathog..

[72] Regla-Nava J.A., Nieto-Torres J.L., Jimenez-Guardeno J.M., Fernandez-Delgado R., Fett C., Castano-Rodriguez C., Perlman S., Enjuanes L., DeDiego M.L. (2015). Severe acute respiratory syndrome coronaviruses with mutations in the E protein are attenuated and promising vaccine candidates. J. Virol..

[73] Almazan F., DeDiego M.L., Sola I., Zuniga S., Nieto-Torres J.L., Marquez-Jurado S., Andres G., Enjuanes L. (2013). Engineering a replication-competent, propagation-defective Middle East respiratory syndrome coronavirus as a vaccine candidate. mBio.

[74] Gutierrez-Alvarez J., Honrubia J.M., Fernandez-Delgado R., Wang L., Castano-Rodriguez C., Zuniga S., Sola I., Enjuanes L. (2021). Genetically Engineered Live-Attenuated Middle East Respiratory Syndrome Coronavirus Viruses Confer Full Protection against Lethal Infection. mBio.

[75] Gutierrez-Alvarez J., Honrubia J.M., Sanz-Bravo A., Gonzalez-Miranda E., Fernandez-Delgado R., Rejas M.T., Zuniga S., Sola I., Enjuanes L. (2021). Middle East respiratory syndrome coronavirus vaccine based on a propagation-defective RNA replicon elicited sterilizing immunity in mice. Proc. Natl. Acad. Sci. USA.

[76] Zhang X., Liu Y., Liu J., Bailey A.L., Plante K.S., Plante J.A., Zou J., Xia H., Bopp N.E., Aguilar P.V. (2021). A trans-complementation system for SARS-CoV-2 recapitulates authentic viral replication without virulence. Cell.

[77] Ju X., Zhu Y., Wang Y., Li J., Zhang J., Gong M., Ren W., Li S., Zhong J., Zhang L. (2021). A novel cell culture system modeling the SARS-CoV-2 life cycle. PLoS Pathog..

[78] Cheung P.H., Ye Z.W., Lui W.Y., Ong C.P., Chan P., Lee T.T., Tang T.T., Yuen T.L., Fung S.Y., Cheng Y. (2022). Production of single-cycle infectious SARS-CoV-2 through a trans-complemented replicon. J. Med. Virol..

